# Identification of risk factors for disseminated cryptococcosis in non-hiv patients: a retrospective analysis

**DOI:** 10.1186/s40001-023-01592-8

**Published:** 2023-12-19

**Authors:** Fan Zhang, Yiqing Zhou, Xiaoqi Tang, Minghui Li

**Affiliations:** 1 School of Medicine, Shaoxing University, Shaoxing, 312000 Zhejiang People’s Republic of China; 2 Department of Infectious Disease, Shaoxing People’s Hospital, 568 Zhongxing Road, Shaoxing, 312000 China

**Keywords:** Pulmonary cryptococcosis, Cryptococcal meningoencephalitis, Disseminated cryptococcosis, Risk factor

## Abstract

**Objective:**

This study aimed to investigate the potential risk factors associated with disseminated cryptococcosis in HIV-negative individuals.

**Methods:**

A total of 106 HIV-negative patients with cryptococcal disease were enrolled. The observation group consisted of patients with disseminated cryptococcosis (DC), whereas the control groups included patients with pulmonary cryptococcosis (PC) and cryptococcal meningitis (CM). Univariate and multivariate logistic regression algorithms were used to explore the significant clinical and laboratory characteristics that affect the progression of cryptococcal infections. Finally, receiver operating characteristics (ROC) curves are applied to assess the diagnostic value of identified risk factors.LE: Kindly check the edit made in the title.I agree

**Results:**

Of the 106 patients, 57 were diagnosed with pulmonary cryptococcosis, 22 with cryptococcal meningitis, and 27 with disseminated cryptococcosis. The logistic regression equation included five variables: diabetes, decompensated liver cirrhosis, long-term use of immunosuppressive agents, decreased serum albumin level, and elevated plasma cytokine IL-10 level. The ROC curves showed that albumin (AUC > 0.7), IL-10 (AUC > 0.7) and decompensated liver cirrhosis (AUC > 0.6) have relatively high diagnostic capacity in predicting the progression of Cryptococcus.

**Conclusion:**

This study identified elevated IL-10 levels as an independent risk factor for developing disseminated cryptococcosis in the control groups. Furthermore, decompensated liver cirrhosis and decreased serum albumin independently affected the progression of cryptococcosis in the CM and PC groups, respectively.

## Introduction

Cryptococcal infection is a prevalent opportunistic fungal infection ranging from asymptomatic lung colonization to life-threatening meningitis and disseminated infections. Although it is commonly observed in individuals with compromised immune function worldwide, variations in patient characteristics and clinical manifestations of the infection exist. Cryptococcal disease is most commonly associated with HIV infections. However, studies have shown that 10–40% of HIV-negative patients with cryptococcal disease lack apparent immune deficiencies and can still be infected with *Cryptococcus* species [1]. Recent research has estimated approximately 250,000 cases of cryptococcal meningitis and 181,000 deaths annually worldwide [2]. Due to limited clinical research on HIV-negative patients with cryptococcal infection, this study aimed to analyze the laboratory and clinical characteristics of patients with disseminated cryptococcosis to investigate the risk factors associated with the disease progression.

## Subjects and methods

### Study participants

A total of 106 hospitalized patients diagnosed with pulmonary cryptococcosis, cryptococcal meningoencephalitis, or disseminated cryptococcosis between January 2016 and March 2023 at the Shaoxing People’s Hospital were included in the study. The diagnostic criteria were adapted from the updated Clinical Practice Guidelines for Cryptococcal Disease in China (2010) by the Infectious Diseases Society of America.

### Data collection

The electronic medical record system of our hospital was used to identify cases with discharge diagnoses of pulmonary cryptococcosis, cryptococcal meningoencephalitis, or disseminated cryptococcosis. The medical records of these patients were reviewed to collect information on the clinical manifestations, relevant clinical data, radiographic findings, laboratory tests, and pathology. The collected data were then organized and summarized.

### Statistical analysis

Statistical analysis was performed using SPSS 25.0 software. The chi-square or Fisher’s exact test was used to compare categorical variables between groups. The mean ± standard deviation (x ± s) was computed for continuous variables, and one-way analysis of variance (ANOVA) was employed to compare groups. Multivariate analysis was performed using logistic regression models and plotted ROC curves. Statistical significance was set at *P* < 0.05.

## Results

### Comparison of basic clinical characteristics and presentations among the three groups of cryptococcal infections

Of the 106 patients, 57 were diagnosed with pulmonary cryptococcosis (PC), 22 had cryptococcal meningitis (CM), and 27 presented with disseminated cryptococcosis (DC). In all three groups, the prevalence was higher among the men than the women. There were no significant differences between the groups regarding gender, age, and hypertension status (*P* > 0.05). However, diabetes, decompensated liver cirrhosis, and long-term use of immunosuppressants exhibited a significantly higher prevalence in the disseminated cryptococcosis group (*P* < 0.05).The most common clinical presentations were cough (43.9%) and sputum production (38.6%) in the PC group, headache (45.5%) and fever (18.2%) in the CM group, and cough (40.7%), followed by fever (37.0%) in the DC group. There were no significant differences in clinical presentations among the three groups (P > 0.05), as shown in Table 1.

**Table 1:** Comparison of basic data and clinical manifestations of patients in the three groups

Variables	PC group (*n* = 57)	CM group (*n* = 22)	DC group (*n* = 27)	χ^2^/F	*P*
Age(mean ± SD)	56.89 ± 13.36	57.36 ± 11.81	51.26 ± 12.80	2.016	0.138
Gender(male)	39(68.4)	15(68.2)	17(63.0)	0.256	0876
Underlying disease					
Hypertension	19(33.4)	5(22.7)	12(44.4)	2.571	0.277
Diabetes mellitus	10(17.5)	1(4.5)	9(33.3)	6.704	0.035
Renal failure	1(1.8)	3(13.6)	1(3.7)	4.274	0.056
Malignant tumor	2(3.5)	1(4.5)	2(7.4)	0.976	0.828
Chronic lung disease	2(3.5)	1(4.5)	3(11.1)	2.048	0.400
Decompensation of cirrhosis	11(19.3)	1(4.5)	9(33.3)	6.344	0.042
Glucocorticoid	4(7.0)	5(22.7)	4(14.8)	3.952	0.135
Immunosuppressant	6(10.5)	2(9.1)	9(33.3)	6.985	0.028
Clinical manifestation					
Fever	15(26.3)	4(18.2)	10(37.0)	2.236	0.327
Cough	25(43.9)	4(18.2)	11(40.7)	4.594	0.101
Expectoration	22(38.6)	3(13.6)	9(33.3)	4.565	0.102
Chest pain	3(5.3)	1(4.5)	1(3.7)	0.289	0.999
Headache	15(26.3)	10(45.5)	5(18.5)	4.574	0.112
Dyspnea	6(10.5)	1(4.5)	4(14.8)	1.283	0.574
Altered state of consciousness	1(1.8)	3(13.6)	2(7.4)	4.446	0.073

Data presented as n of patients (%)

### Comparison of laboratory parameters among the three groups of *Cryptococcus*-infected cases

The CM group showed higher white blood cell counts and neutrophil proportion than the PC and DC groups; however, the differences were insignificant (*P* > 0.05). The DC group had significantly lower serum albumin and higher IL-10 levels than PC and CM groups (*P* < 0.05). However, there were no significant differences in the other laboratory parameters among the groups (*P* > 0.05), as shown in Table 2.

**Table 2:** Comparison of laboratory test results among the three groups

Variables	PC group (n = 57)	CM group (*n* = 22)	DC group (*n* = 27)	χ^2^/F	*P*
WBC (× 109/L)	5.97± 1.99	7.17± 3.40	7.19± 4.07	2.178	0.119
NE %	69.35± 8.05	72.09± 13.92	72.99± 8.73	1.523	0.223
EOS %	4.591± 1.53	4.323± 1.33	4.463± 1.50	0.273	0.762
Alb (g/L)	38.92± 5.92	38.69± 3.94	35.04± 3.89	5.674	0.005
CRP (mg/L)	13.84± 22.66	12.22± 26.40	16.72± 25.28	0.227	0.797
CD4 + T %	35.27± 10.96	35.53± 8.79	29.86± 8.93	2.985	0.055
CD8 + T %	15.93± 5.48	17.74± 5.12	15.81± 4.16	1.152	0.320
IL-4 (pg/mL)	6.53± 3.45	6.91± 2.98	7.19± 1.87	0.918	0.428
IL-10 (pg/mL)	3.19± 2.35	2.90± 1.24	5.06± 2.97	7.023	0.001

Data presented as mean ± SD; WBC, white blood cell; NE, neutrophils; EOS, eosimophil; Alb, albumin; CRP, C-reactive protein; L-10, Interleukin-10

### Diagnostic methods, treatment approaches, and prognosis analysis of *Cryptococcus*-infected patients

The infection rates were 53.8% in the PC group, 20.8% in the CM group, and 25.5% in the DC group. The PC group primarily relied on pathological examinations and blood cryptococcal capsular antigen testing for diagnosis. The CM group utilized cerebrospinal fluid smear or culture and cerebrospinal fluid cryptococcal capsular antigen testing. The DC group also relied on pathological examinations and blood cryptococcal capsular antigen testing for diagnosis. In the DC group, the most frequent presentation was the simultaneous involvement of the central nervous system (CNS) and lungs (37.0%), followed by the concurrent involvement of the lungs and skin (33.4%). The patients received predominantly medical treatment alone or combined with surgical interventions. The mortality rates were 5.3% in the PC group, 9.0% in the CM group, and 25.9% in the DC group, as shown in Table 3.

**Table 3:** Diagnostic methods, treatment methods and prognosis of patients with cryptococcus infection

Sites of involvement	N	Diagnostic method						Death
		Pathological examination*	CSF smear or culture	Blood or tissue culture	CrAg (Blood)	CrAg (CSF)	Therapies	
Pulmonary	57	48	—	17	23	—	Drug ± surgery	3
Intracranial	22	—	18	—	—	19	Drug	2
Pulmonary and intracranial	10	6	4	1	—	4	Drug ± surgery	4
Pulmonary and skin	9	5	—	2	8	—	Drug ± surgery	1
Pulmonary and blood	1	—	—	1	1	—	Drug	1
Pulmonary and throat	1	1	—	—	1	—	Drug ± surgery	0
Pulmonary and ribs	1	1	—	—	1	—	Drug	0
Pulmonary and heart	1	1	—	—	—	—	Drug ± surgery	0
Pulmonary and intracranial and blood	2	1	1	1	—	—	Drug	1
Pulmonary and intracranial and skin	1	1	—	—	1	1	Drug	0
Pulmonary and waist and blood	1	1	—	1	1	—	Drug	0
Sum up	106	65	23	23	36	24	—	12

^*^Includes lung puncture/surgical biopsy, skin biopsy, muscle biopsy; *CrAg* cryptococcal capsular polysaccharide antigen, *CSF* cerebrospinal fluid

### Comparison of pulmonary computed tomography (CT) imaging between the PC and DC groups

In the PC group, the lesions were primarily observed in one lung (49.1%), and solitary lesions were more frequent (61.4%). Conversely, bilateral lung involvement was predominant (59.3%) in the DC group, with a higher occurrence of multiple lesions (66.7%). There was a significant difference in the extent of lesions between the two groups (P < 0.05); however, no such differences were observed in CT imaging characteristics between the two groups. The nodular or mass-like patterns were the most commonly observed, followed by patchy or ground-glass opacities. These patterns were frequently accompanied by cavitation, halo sign, and pleural effusion, as shown in Table 4.

**Table 4:** Pulmonary CT imaging findings in the PC and DC groups

Item		PC group (*n* = 57)	DC group (*n* = 27)	χ^2^	*P*
Extent of disease	Unilateral	28(49.1)	11(40.7)	0.518	0.472
	Bilateral	19 (33.3)	16 (59.3)	5.067	0.024
	solitary	35 (61.4)	9 (33.3)	5.788	0.016
	multiple	22 (38.6)	18 (66.7)	5.788	0.016
Imaging findings	Nodular mass type	30 (52.6)	16 (59.3)	0.325	0.569
	Patchy shadow or ground glass infiltration shadow	23 (40.4)	13 (48.1)	0.455	0.500
	Halo sign	6 (10.5)	4 (14.8)	0.321	0.720
	Cavity	10 (17.5)	8 (29.6)	1.589	0.207
	Pleural effusion	5 (8.8)	4 (14.8)	0.699	0.460
	The pleura was thickened	4 (7.0)	3 (11.1)	—	0.676
	Air bronchogram sign	3 (5.3)	2 (7.4)	—	0.655
	Lymph nodes	2 (3.5)	2 (7.4)	—	0.591

### Independent risk factors for disseminated cryptococcosis

The statistically significant factors (decompensated liver cirrhosis, diabetes, long-term use of immunosuppressants, serum albumin, IL-10) represented in Table 1 were included in the logistic regression model for multivariate analysis. The results showed that elevated IL-10 was an independent risk factor for disseminated cryptococcal infection in the PC group and CM group, while a decrease in serum albumin and decompensated cirrhosis were an independent risk factor for disseminated cryptococcal infection in the PC group and the CM group, respectively, as shown in Table 5.

**Table 5:** Multivariate regression analysis of disseminated cryptococcal infection

Variables		B	SE	Wald/χ^2^	*P*	OR	95%CI
Diabetes mellitus	PC group	0.706	0.649	1.185	0.276	2.026	0.568–7.225
	CM group	2.300	1.205	3.644	0.056	9.970	0.940–105.712
Decompensation of Cirrhosis	PC group	1.242	0.691	3.224	0.073	3.461	0.893–13.423
	CM group	3.092	1.243	6.190	0.013	22.012	1.927–251.413
Immunosuppressant	PC group	1.179	0.693	2.892	0.089	3.250	0.836–12.639
	CM group	1.336	0.969	1.898	0.168	3.802	0.569–25.421
Alb	PC group	0.116	0.056	4.306	0.038	1.123	1.006–1.252
	CM group	0.116	0.068	2.890	0.089	1.123	0.982–1.285
IL-10	PC group	− 0.335	0.112	8.969	0.003	0.715	0.574–0.891
	CM group	− 0.509	0.169	9.049	0.003	0.601	0.431–0.837

### ROC evaluation

**Fig. 1:**
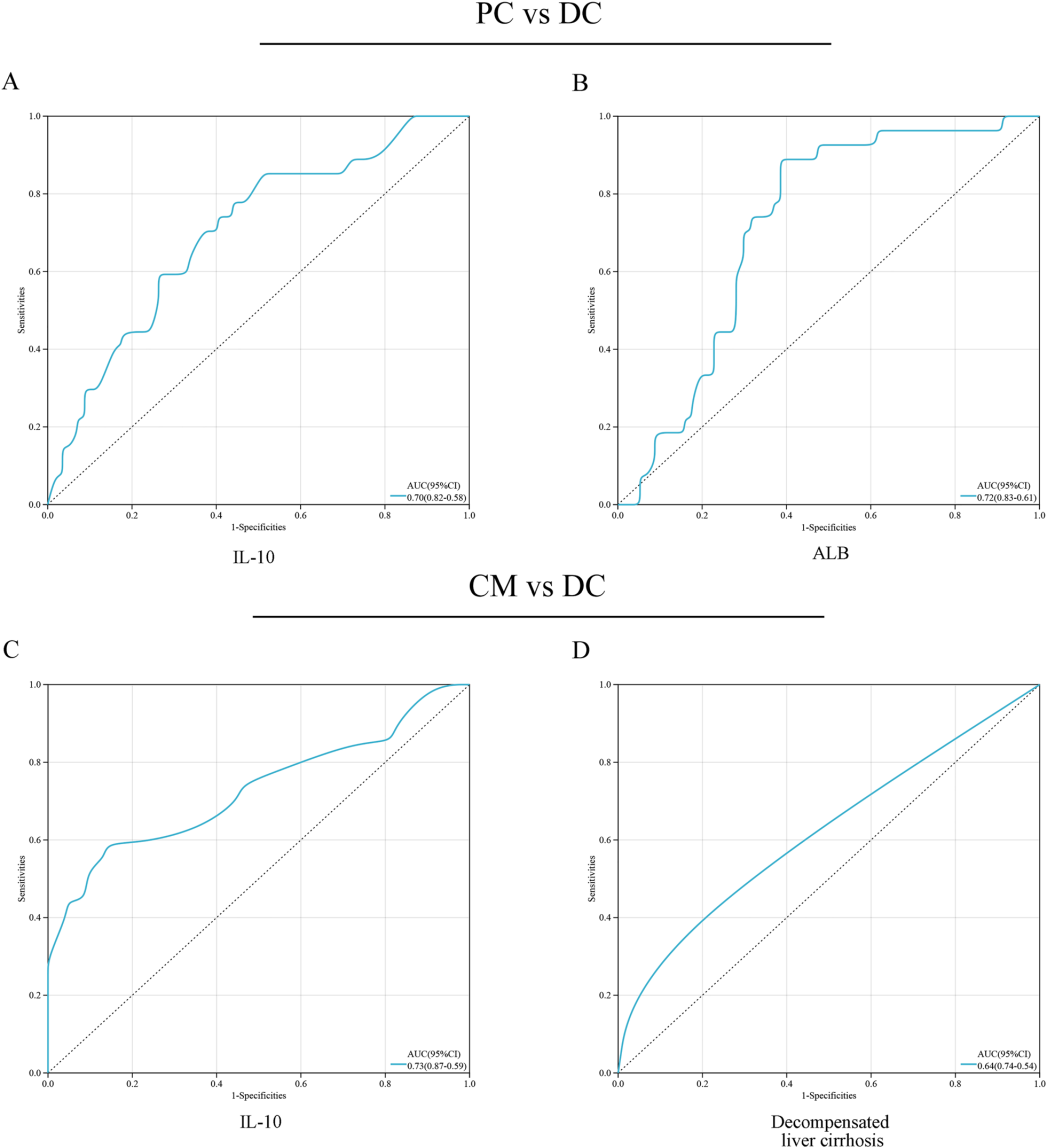
Receiver operating characteristic (ROC) curves for identifying cryptococcus progression. **A**, **B** The ROC curves of 2 variables (IL-10 and ALB) in PC versus DC groups. **C**, **D** The ROC curves of 2 variables (IL-10 and Decompensated liver cirrhosis) in CM versus DC groups. *IL-10* interleukin 10, *ALB* albumin, *PC* pulmonary cryptococcosis, *DC* disseminated cryptococcosis, *CM* cryptococcal meningitis

In the ROC curve, the AUC values corresponding to IL-10 and albumin are 0.701 (95% CI 0.583 0.820) and 0.719 (95% CI 0.608 0.829) (Fig. 1A, B), indicating that these two laboratory parameters have significant value in predicting the progression of PC. The corresponding AUC values for IL-10 and decompensated liver cirrhosis were 0.730 (95% CI 0.589–0.870) and 0.644 (95% CI 0.490–0.798) (Fig. 1C, D), indicating that these two laboratory parameters have significant value in predicting the progression of DM to DC. Detailed information of the ROC parameters is listed in Table 6.

**Table 6:** ROC curve analysis of risk factors affecting the progression of cryptococcus

Group	Variable	AUC	SE	P	95%CI	Cut-off value	Youden index(Max)	
							Sensitivity	Specificity
PC vs DC	IL-10	0.701	0.060	0.003	0.583–0.820	2.325	0.852	0.491
	ALB	0.719	0.056	0.001	0.608–0.829	38.195	0.614	0.889
CM vs DC	IL-10	0.730	0.072	0.006	0.589–0.870	3.885	0.593	0.864
	Decompensation of Cirrhosis	0.644	0.079	0.086	0.490–0.798	–	–	–

*PC* pulmonary cryptococcosis, *DC* disseminated cryptococcosis, *CM* cryptococcal meningitis

## Discussion

Cryptococcal infection is primarily associated with HIV infection, with an annual incidence of cryptococcal meningitis ranging from 5 to 10% among HIV-positive patients [3]. However, it is noteworthy that patients with malignancies, organ or stem cell transplants, severe diabetes, and autoimmune diseases are also prone to being affected by Cryptococcus, leading to a rising incidence of cryptococcal infection worldwide. In China, most cryptococcal infections occur in HIV-negative patients [4]. Current research suggests that the susceptibility to cryptococcal infection in the healthy population in China may be associated with specific genetic factors, such as polymorphisms in genes such as FCGRIIB (Fc gamma receptors IIB) and Dectin-2, as well as gene defects in mannose-binding lectin (MBL). Surawut et al. [5] found that in FCGRIIB ^−/−^mice, the transmissibility of Cryptococcus is enhanced via the trojan horse mechanism, and its gene functional polymorphism is considered a novel contributing factor for acquiring Cryptococcus. Kitai et al. [6] revealed that Dectin-2 gene knock-out can significantly reduce the capability pulmonary macrophages to engulf Cryptococcus neoformans. Additionally, MBL plays an extremely crucial role in the body's defense against pathogen invasion, including cryptococcus [7].

The average age of the included patients was 56 years, with a majority of males (male-to-female ratio of 2.42:1). Male participants were primarily engaged in physical labor, which increased their exposure to Cryptococcus through contacting with soil, dust, and other sources [8]. Prolonged alcohol consumption, smoking, and staying up late also contribute to the risk of Cryptococcus infection. All enrolled patients were residents in subtropical regions, where warm and humid climates are conducive to the growth and reproduction of Cryptococcus [9]. The CNS and lungs are the organs most commonly affected by cryptococcosis [10]. In this study, patients with disseminated cryptococcosis frequently exhibited simultaneous involvement of multiple sites, with the CNS and lungs being the most prevalent, followed by the lungs and skin. Disseminated cryptococcosis carries a high mortality rate and poor prognosis. By analyzing patient information, laboratory test results, clinical manifestations, and pathological findings of patients in the PC, CM, and DC groups, our study aims to identify the risk factors for disseminated cryptococcosis. Early identification of these risk factors could guide healthcare professionals in implementing timely and proactive interventions, ultimately improving patient outcomes.

In the present study, the mortality rate in the DC group was significantly higher than in the PC and CM groups. The multivariate logistic regression analysis showed that elevated plasma cytokine IL-10 levels independently contributed to disseminated cryptococcosis in both PC and CM groups. Previous studies have demonstrated that neutrophils are critical in host defense against cryptococcal infection. Neutrophil recruitment to *Cryptococcus* species requires activation of the complement C5a-C5aR pathway and activation of extracellular signal-regulated kinase (ERK) and p38 mitogen-activated protein kinase (MAPK) systems, resulting in the production of pro-inflammatory cytokines, including elevated levels of IL-10 and IL-12 [11]. The cryptococcal polysaccharide glucuronoxylomannan promotes the secretion of IL-10 and IL-4 [12]. Moreover, IL-10 has been associated with cryptococcal evasion in several studies. A Th2 immune response to new *Cryptococcus* strains induces their virulence factors to enhance the release of IL-10. Consequently, IL-10induces a Th2 response while inhibiting the Th1 response [13]. Elevated levels of IL-10 have been associated with uncontrolled fungal infections [14]. Meanwhile, Seagal et al. [15] emphasized that the pathogenicity of Cryptococcus in IL-10-/- mice was weakened, and the activity of monocyte derived DC, T cells, alveolar macrophages, etc. involved in clearing pulmonary Cryptococcus was enhanced in the lungs, indicating the therapeutic potential of IL-10 blockade in the treatment of fungal pulmonary infections.

Accumulated studies suggested that decompensated liver cirrhosis beresponsiblefor developing disseminated cryptococcosis [16]. In a retrospective study [17], patients with liver cirrhosis were more susceptible to encounter cryptococcus. As the Child–Pugh score of liver functions increase, localized cryptococcosis is more likely to develop systemic spread, which mightleadtoincreased mortality in the DC group [18]. We found that decompensated liver cirrhosis is an independent risk factor for developing disseminated cryptococcosis in patients with cryptococcal meningitis. This association could be attributed to *Cryptococcus* species bypassing the liver’s clearance system in patients with decompensated liver cirrhosis and directly entering the systemic circulation through collateral circulation, resulting in cryptococcal sepsis and subsequent dissemination to the CNS [17]. Patients with decompensated liver cirrhosis have impaired innate and cell-mediated immunity, increasing the susceptibility to invasive cryptococcal disease. Moreover, the mortality risk is high with the onset of the disease [19].

Compared with PC group, the serum albumin of patients decreased in the DC group. It is reported that albumin can inhibits the progress of Cryptococcus neoformans by disrupting the stability of fungal extracellular vesicles [20]. Although we did not find a significant difference in albumin between the CM and DC groups, Yu et al. [21] revealed that serum ALB levels may be associated with the mortality rate of cryptococcal meningitis. Therefore, largerstudiesarerequired to explore the correlation between albumin and the occurrence and progression of Cryptococcus.

In the single-factor analysis, diabetes and long-term use of immunosuppressive agents showed significant association with the dissemination of cryptococcal infection. However, the results were inconsistent in the multivariate analysis. This variation could be attributed to the limited sample size, which affects the statistical power. Research has indicated that patients with diabetes experience metabolic abnormalities and compromised immune function, creating favorable conditions for fungal growth and invasion, thereby increasing the risk of fungal infection in the human body [22]. Individuals with diabetes are more susceptible to fungal infections than healthy individuals [23]. Prolonged use of immunosuppressive agents weakens cellular and humoral immunity and increases the vulnerability to fungal infections. *Cryptococcus* species could rapidly disseminate throughout the body via the bloodstream [24].

In addition to the factors mentioned above influencing the risk of disseminated cryptococcosis, new cryptococcal infections are commonly observed in patients on long-term glucocorticoid use, accounting for approximately one-third of HIV-negative patients [25]. The extended use of glucocorticoids reduces the production of pro-inflammatory cytokines and weakens the body’s ability to resist pathogenic microorganisms, thereby increasing the likelihood of fungal infections [26]. Eosinophils contribute to the body's defense against fungal infections, especially by clearing fungi from the lungs. They are commonly associated with toxic mediator release and phagocytic activity [27].

The CT manifestations of pulmonary cryptococcal infections vary widely. When Cryptococcus invades the lungs, it triggers the body,s immune and inflammatory responses, leading to macrophages and multinucleated giant cells engulfing the pathogens to form non-caseating granulomas or connective tissue containing the fungus [28]. Nodules or mass-like lesions are the main manifestations of chest CT scanning in general individuals with cryptococcal infections, while patients with diabetes, malignant tumor, and impaired immunity typically display pneumonia-like or mixed patterns [29]. Consistent with these findings, the PC group in our study had a lower incidence of comorbidities and long-term utilization of immunosuppressive agents, with a higher prevalence of a solitary lung nodular/mass pattern (52.6%). The overall condition of patients in the DC group was more severe, predominantly characterized by patchy bilateral opacities or ground-glass infiltrates (59.3%). Additionally, pulmonary cryptococcal infections can also encounter halo signs, pleural effusion, cavitation, pleural thickening, and bronchial inflation signs. The present study indicated that patients with disseminated cryptococcal infection predispose to more severe pulmonary imaging features, which may contribute to enhanced management of Cryptococcus disease in terms of its progression and prognosis.

Lung biopsy is a common strategy for diagnosing pulmonary cryptococcosis [30]. When lesions are located in the outer lung field, the success rate of lung puncture operation is higher, followed by pathological examination and detection of cryptococcal capsule antigen [31]. For the diagnosis of cryptococcal meningitis, routine cerebrospinal fluid testing, biochemistry, and cryptococcal capsule antigen testing are feasible [32]. If skin is involved, tissue culture combined with skin biopsy can be employed to improve detection accuracy [33]. Among 106 patients with cryptococcal diseases, 80 were treated with medication alone and other 26 were treated with surgery combined with medication. An antibiotic strategy maintaining 12 to 18 months is used to treat severe pulmonary cryptococcosis or combined with central nervous system infection, which start with amphotericin B combined with 5-fluorocytosine, followed by fluconazole treatment. If diagnosed with intracranial infection, it is recommended to perform ventriculoperitoneal shunt [34]. For postoperative patients with pulmonary cryptococcosis, if no abnormalities are found in clinical symptoms, immune function, serology and imaging examinations, and indications of extrapulmonary infection are ruled out, the guidelines suggest that antifungal drugs may not be necessary. In the PC group, 5 patients developed respiratory symptoms after surgery and discovered new lesions by antigen testing and imaging verification, which possibly due to the recurrence of pulmonary cryptococcus. Ultimately, these patients recovered after treatment with fluconazole. Given the different conditions of the lesion and surgical procedures, postoperative antifungal therapy may be beneficial for preventing postoperative recurrence. Furthermore, one patient initially exhibited only mild symptoms with a headache, and the diagnosis was not clear until disseminated Cryptococcus lesions were detected. Two patients were misdiagnosed as pulmonary tuberculosis or tumors in the early stage, resulting in delayed treatment, ultimately leading to the dissemination and development of Cryptococcus. Therefore, early detection of Cryptococcus capsular antigen can effectively prevent trauma caused by surgery or biopsy, and potentially reducing the disease severity and the risk of death [35].

The advantages of this study include the application of computerized in-patient registration and using B45 diagnostic codes to identify all cases of cryptococcal infection during study period, as well as the validation of diagnosis by reviewing medical records and laboratory and microbiological tests. In addition, information on potential risk factors is systematically collected from medical information, this approach avoided patient recall bias common in case–control studies. However, the limitations of this study should be acknowledged. This is a single-center study with a limited number of cases included. Meanwhile, due to the retrospective nature of this study, some clinical data are incomplete, may limit the statistical validity. For example, many patients did not have cryptococcal capsule antigen testing, so it fails to be included in the analysis of potential risk factors. Furthermore, the limited follow-up data hamper the further investigation of these identified factors affecting prognosis. Thus, larger scale multicenter randomized controlled trials are needed to testify our viewpoint.

In summary, *Cryptococcus* species potentially invade various tissues and organs in the human body, posing a significant health threat. Pulmonary cryptococcosis is the most prevalent form, characterized by a fungal infection of the respiratory system resulting from inhalation of cryptococcal spores. Cryptococcal infections can spread from the lungs to the CNS and other extrapulmonary sites. With the continuous evolution of *Cryptococcus* species and an increasingly susceptible population, disseminated cryptococcosis cases are increasing. In our study, the PC group exhibited a more favorable prognosis than the CM and DC groups, with the highest mortality rate observed in the DC group, followed by the CM group. Elevated IL-10 level was identified as an independent risk factor for disseminated cryptococcosis in both the PC and CM groups. In contrast, decompensated liver cirrhosis was identified as an independent risk factor for disseminated cryptococcosis in the CM group. Decreased serum albumin level was identified as an independent risk factor for disseminated cryptococcosis in the PC group. Therefore, patients with disseminated cryptococcosis must remain highly vigilant. When PC and CM patients present elevated IL-10decreased serum albumin, and decompensated cirrhosis, the likelihood of disseminated infection increases. Therefore, clinical physicians should assess disseminated infections in advance based on risk factors, and take appropriate prevention and treatment measures to reduce the mortality rate of Cryptococcus.

## Data Availability

The data used to support the findings of this study are included within the article.
